# Anthocyanin-Loaded Double Pickering Emulsion Stabilized by Phosphorylated Perilla Seed Protein Isolate–Pectin Complexes and Its Environmental Stability

**DOI:** 10.3390/foods14091650

**Published:** 2025-05-07

**Authors:** Zhao Chen, Jun Yang, Hao Guo, Xiuling Zhang, Wentao Zhang

**Affiliations:** College of Food Science, Northeast Agricultural University, Harbin 150030, China; chenzhao200003@163.com (Z.C.); yj13684515276@126.com (J.Y.); 13304633979@163.com (H.G.)

**Keywords:** phosphorylated perilla seed protein isolate, double Pickering emulsion, thermal stability, ionic strength, freeze–thaw cycle

## Abstract

Thus far, the focus of research has been on employing perilla seed protein isolate (PSPI) to stabilize emulsions encapsulating hydrophobic substances, but there is a dearth of studies focusing on PSPI-stabilized double emulsions for encapsulating hydrophilic materials. This experiment investigated the environmental stability (thermal, ionic strength, and freeze–thaw stability) of PSPI-stabilized double emulsions encapsulating anthocyanins. During thermal stability experiments, the emulsion color lightened as the treatment temperature increased, whereas the microstructures of the emulsions exhibited no notable differences among the groups. The anthocyanin retention and antioxidant capacity decreased with increasing thermal treatment temperature. After thermal treatment, no creaming or separation was observed, and anthocyanin retention remained above 65% in all groups. Ionic strength exerted a certain influence on the stability of the emulsions, with droplet size increasing and anthocyanin retention dwindling as ionic strength intensified. At an ionic strength of 100 mmol/L, anthocyanin retention surpassed 70%. No delamination was observed at any of the ionic strengths. With the augmentation of freeze–thaw cycles, the emulsions darkened and yet remained unseparated, droplet size progressively increased, and anthocyanin retention progressively decreased. The findings indicate that the emulsions were environmentally stable and could serve as a reference for the development of related emulsions.

## 1. Introduction

A double emulsion (W_1_/O/W_2_ emulsion) is a multiphase system characterized by its unique “two membranes and three phases” structure. Since William Seifriz discovered their W/O/W structure and proposed the concept of a “double emulsion” in 1925, double emulsions have gradually been researched and applied. In the early stages, because of their structural complexity and instability, double emulsions did not attract much attention. However, with the successful preparation of insulin-containing double emulsions by Engel et al. in 1968 [1], the exploration of double-emulsion applications officially commenced. In the 21st century, the prospects for the application of double emulsions in pharmaceuticals, food, skin care, and polymers are growing more promising because of in-depth research on their structures. The ability of double emulsions to not only isolate and control the release of their components, but also to contain different substances in different phases creates nearly unlimited possibilities for their use in many areas [2,3]. Meanwhile, compared with O/W emulsions, W/O/W (water-in-oil-in-water) emulsions have a lower fat content. This characteristic makes them more aligned with the current dietary trend of seeking health and low-fat options [4].

The versatility of double emulsions is mainly due to the designability of their structures and the advantages of encapsulating diverse active substances. In the pharmaceutical field, double emulsions can be used for drug encapsulation and controlled release to enhance the stability and bioavailability of medications; in the food industry, double emulsions can be used to enhance the palatability and durability of food products and prolong shelf life; and in skin-care and chemical fields, double emulsions can be used to prepare cosmetic and chemical products with special functions [5,6,7,8,9]. Recently, double Pickering emulsions have emerged as a research focus owing to their distinctive advantages. These systems utilize solid particles to stabilize single or dual interfaces, combining the encapsulation capacity of conventional double emulsions with the characteristic stability and low toxicity of Pickering emulsions, demonstrating significant potential for controlled-release delivery and food preservation applications [3,10]. Pan et al. [11] developed anthocyanin-loaded double Pickering emulsions stabilized by β-cyclodextrin, which demonstrated excellent stability under both centrifugal and thermal conditions. María Matos et al. [12] stabilized double Pickering emulsions using quinoa starch particles, with the initial encapsulation efficiency of the prepared emulsions reaching approximately 95%.

Perilla is used as a traditional Chinese herbal medicine and food ingredient [13]. However, perilla seed protein resources have not yet been rationally and effectively utilized. Defatted perilla meal derived from perilla seeds boasts a high protein content, and this protein has good emulsifying activity. The bioactive properties of perilla protein have been preliminarily established, with research showcasing its efficacy in combating fatigue, bolstering reproductive health, mitigating high blood pressure, managing diabetes, and neutralizing oxidative stress [14]. In the study of Ja Min Kim et al. [15] they obtained antioxidant peptides from perilla seed meal (PSM). The peptide showed good antioxidant activities such as reducing power, DPPH radical scavenging activity and ABTS radical scavenging activity. A low molecular weight peptide, which was obtained from PSM protein by Bo Yeon Park et al. [16], could inhibit the activities of α-glucosidase and angiotensin-I-converting enzyme, and thus play an antidiabetic and antihypertensive role. In recent years, in-depth research on perilla seed proteins has made the prospects for their application in emulsifiers increasingly promising. However, the current research on perilla proteins mainly focuses on their extraction, functional properties, and application in O/W emulsions. For example, Zhao et al. [17] used phosphorylated perilla protein isolate (LZPI) to prepare high-internal-phase emulsions loaded with β-carotene. Furthermore, regarding double emulsions stabilized by perilla seed protein, there is still a research gap concerning their stability when exposed to environmental conditions [18,19].

In the food industry, pectin (PEC), as a natural emulsifier, has a wide range of applications [20,21]. Pectin emulsifiers can reduce the separation of fats and oils in water and improve the stability and texture of food products to extend their shelf lives. Pectin’s natural and safe properties make it an important additive in food products such as candies, jellies, breads, and dairy products. The application of pectin emulsifiers not only improves the quality of food products but also brings consumers a more delicate, soft, and lubricating taste experience [22]. Sahar Abdoollahi et al. [23] devised an egg-free mayonnaise recipe by incorporating chia seed protein hydrolysate (CSPH) and pectin extracted from apple pomace (PA). Their study revealed that replacing egg with CSPH and PA not only enhanced the mayonnaise’s acidity but also bolstered its physical and thermal stability, viscosity, hardness, and adhesiveness. This innovative formulation offers a viable alternative for mayonnaise enthusiasts with egg allergies.

In addition, protein–polysaccharide complexes also offer unique advantages as emulsifiers [24,25]. Protein–polysaccharide complexes have multiple adsorption sites that can be simultaneously immobilized at the oil–water interface to form a viscoelastic film and improve the spatial stability of emulsions. Wang et al. [26] demonstrated that Sanxan, a novel microbial polysaccharide, markedly improved emulsification activity compared to proteins alone. This was attributed to Sanxan exposing protein hydrophobic groups, thereby enhancing oil droplet stabilization. As an amphiphilic polysaccharide, Sanxan further contributed to emulsification. Han et al. [27] revealed that the interaction between myofibrillar protein (MP) hydrophilic groups and high methoxyl pectin (HMP) elevated the hydrophilicity of the MP–HMP complex. In the MP–HMP oil–water system, amphiphilic MP quickly formed an interface, and hydrophilic HMP enhanced interfacial properties, thereby preventing oil leakage and MP aggregation. Additionally, HMP integration increased MP adsorption on droplet surfaces, improving emulsifying stability. Therefore, such complexes are also promising for applications in areas such as food and pharmaceuticals. By means of non-covalent or covalent binding, proteins and polysaccharides can interact with each other to form a stable emulsion layer, which improves the physical and rheological properties of emulsions [28,29]. In this experiment, the complex of phosphorylated perilla seed protein isolate (PPSPI) and PEC will be used as an emulsifier. At pH 3.0, perilla seed protein isolate (PSPI) carries a positive charge and forms a complex with negatively charged PEC through electrostatic interaction [18,30,31,32]. This complex is expected to have improved emulsifying properties compared to PPSPI alone due to the following reasons. Firstly, compared to using PPSPI alone, the addition of PEC can create steric hindrance among protein molecules, effectively reducing protein aggregation and enabling more uniform and stable dispersion of proteins at the oil–water interface [31]. Secondly, PPSPI exhibits highly hydrophobic properties and limited hydrophilicity; the introduction of the hydrophilic molecule PEC will endow the complex of the two substances with both hydrophilic and lipophilic properties, thereby stabilizing the emulsion more effectively [33,34]. Additionally, PEC itself possesses certain emulsifying properties, which enhance emulsion stability [32].

Anthocyanin, a powerful antioxidant, offers multiple bioactive functions, such as anti-inflammatory effects [35], vision protection, antioxidant properties, cardiovascular health benefits, and enhancement of physical fitness. These functions make anthocyanins valuable for broad applications in food, nutraceuticals, and cosmetics. However, anthocyanins are unstable under light, heat, and metal ion conditions. Therefore, it is crucial to employ double emulsions for encapsulating anthocyanins to expand their applications in emulsion systems and enhance their stability [11,36]. Currently, there are studies on the encapsulation of anthocyanins using double emulsions. For example, in the research conducted by Huang et al. [37] and Lin et al. [38], the encapsulation of anthocyanin compounds in double emulsions was achieved by utilizing genipin-crosslinked alkaline-soluble polysaccharide–whey protein isolate conjugates and octenylsuccinate quinoa starch, respectively. Furthermore, it was observed that the encapsulation efficiency in both investigations exceeded 90%; however, an assessment of the emulsions under different environmental conditions was lacking.

Double Pickering emulsions, as an innovative delivery system, demonstrate remarkable advantages over conventional emulsions. Their solid particle-stabilized interfacial architecture forms a denser physical barrier that significantly enhances resistance to coalescence and Ostwald ripening, and the unique “two-phase three-membrane” structure enables co-encapsulation and the controlled release of bioactive compounds having different properties [39]. To address the underutilization of PSPI, a high-quality plant protein resource, this study innovatively employs PPSPI in combination with PEC as a stabilizer for double Pickering emulsions. The phosphorylation treatment enhances the interfacial activity of PSPI [40,41,42]. The resulting PPSPI–PEC complex, formed through electrostatic interactions, serves as an excellent emulsifier: the protein component delivers outstanding emulsification performance, while pectin significantly improves emulsion stability through steric hindrance and thickening effects [32]. This natural emulsification system utilizing agricultural byproducts presents a sustainable solution that simultaneously addresses resource utilization challenges and offers innovative approaches for functional food development.

This study presents new double Pickering emulsion (W_1_/O/W_2_ Pickering emulsion) formulations that can be used to encapsulate hydrophilic actives. In this study, we prepare a double Pickering emulsion with a PPSPI–PEC complex as the external emulsifier and investigate the environmental stability of the W_1_/O/W_2_ Pickering emulsion, as well as the protective effect of this emulsion on anthocyanins.

## 2. Materials and Methods

### 2.1. Materials

Defatted perilla seed flour and perilla seed oil were provided by Huanan Nongshengyuan Food Co., Ltd. (Jiamusi, China). Polyglyceryl-3 polyricinoleate (PGPR) and pectin (PEC) from citrus were obtained from Yuanye Co., Ltd. (Shanghai, China). Anthocyanins from blue honeysuckle were supplied by Heilongjiang Senlan Berry Technology Co., Ltd. The 1,1-diphenyl-2-picryl-hydrazyl radical (DPPH) was purchased from Beijing Boao Tuoda Technology Co., Ltd. The 2, 2′-azino-bis (3-ethylbenzothiazoline-6-sulfonic acid) (ABTS) was obtained from Solarbio Biochemical Co., Ltd. (Beijing, China). All other chemical reagents used were analytical grade.

Dialysis membranes (molecular weight cut-off 8000 Da) were purchased from Beijing Boao Tuoda Technology Co., Ltd (Beijing, China).

### 2.2. Preparation and Characterization of PPSPI–PEC Complexes

#### 2.2.1. Preparation of Perilla Seed Protein Isolate (PSPI)

**Figure 1:**
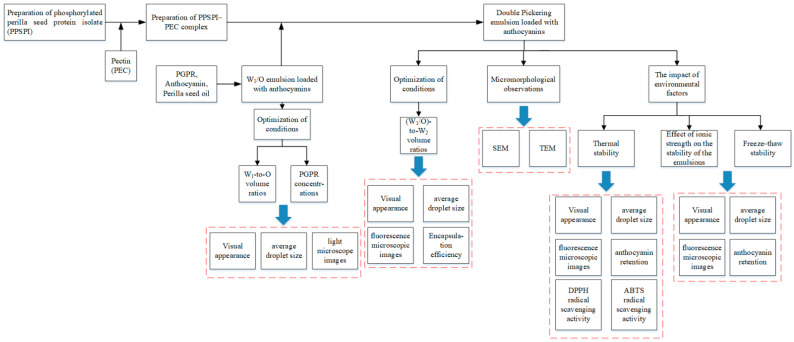
Schematic representation of the research directions. Black arrows in the figure represent the experimental procedure, and blue arrows denote the testing parameters.

Figure 1 illustrates the overall experimental design. PSPI was obtained from defatted perilla seed flour. PSPI was prepared using a modified version of a method given in previous reports [43]. Briefly, the defatted perilla seed flour was dispersed in deionized water (1:15 g/mL). NaOH (1 mol/L) was used to change the pH of the dispersion to 9.0. The mixture was centrifuged for 15 min after constant stirring for 2 h at 45 °C. The pH of the supernatant was changed to 4.5 using HCl (1 mol/L). The protein precipitate was separated via centrifugation and subsequently neutralized by washing it with deionized water. Subsequently, the PSPI was freeze-dried using a JW-FD-18S Lab vacuum freeze dryer (Shanghai JinWen Instrument Co., Ltd.) and stored at 4 °C for future use.

#### 2.2.2. Preparation of Phosphorylated Perilla Seed Protein Isolate (PPSPI)

Based on the methodology reported by Zhao et al. [34], a 0.06 g/mL PSPI dispersion was stirred for 4 h. After overnight hydration at 4 °C, add sodium tripolyphosphate (8%, based on the mass of PSPI) and adjust the pH of the mixture to 9.0. After being incubated for 2 h at 45 °C, the mixture was dialyzed using dialysis membranes and then lyophilized with a JW-FD-18S Lab vacuum freeze dryer (Shanghai JinWen Instrument Co., Ltd.) to obtain PPSPI. Protein purity was determined to be 81.33% using the Coomassie Brilliant-Blue method [43]. The determination of organic phosphorus content was conducted in accordance with the Chinese agricultural standard NY/T 2017–2011 [44]. The samples were decocted with sulfuric acid–hydrogen peroxide. The total phosphorus content was determined using the molybdenum–antimony anti-spectrophotometric method. The inorganic phosphorus content was measured using phosphomolybdate blue spectrophotometry. The organic phosphorus content was determined by ascertaining the difference between the total phosphorus content and the inorganic phosphorus content. The organic phosphorus content of PPSPI was 3416.57 mg/kg, and that of PSPI was 3204.20 mg/kg.

#### 2.2.3. Preparation of PPSPI–PEC Complexes

Based on the methods reported by Li et al. [43] and Zhao et al. [34] (with appropriate modifications), a protein stock solution (0.04 g/mL) was prepared by dispersing the PPSPI. After overnight storage, remove the insoluble materials via centrifugation for 10 min. PEC was solubilized to prepare a pectin stock solution, which was stored overnight at 4 °C. After heating the resulting protein dispersion at 90 °C for 20 min, it was instantly cooled to room temperature. A protein–pectin dispersion was created by mixing the same volumes of PPSPI dispersion and pectin dispersion (0.04 g/mL). Referring to our previous research, after stirring, the pH of the PPSPI–PEC complexes was changed to 3.0 using HCl (1 mol/L). The final concentration of protein in the mixture system was 0.02 g/mL.

### 2.3. W_1_/O/W_2_ Pickering Emulsions

#### 2.3.1. Preparation of W_1_/O Emulsions and Optimization of Conditions

The W_1_/O emulsion was first prepared with a 0.01 g/mL anthocyanin solution, and this solution was referred to as W_1_. Then, 0.5, 1, 3, 5, and 7% (*v*/*v*) PGPR were dissolved in perilla seed oil. Add the W_1_ to perilla seed oil (O) to prepare W_1_/O emulsions at various W_1_-to-O volume ratios (1:9, 2:8, 3:7, 4:6, and 5:5). High-speed shearing was conducted at 15,000 rpm for 5 min using an Ultra-Turrax IKA-T25 device (Staufen, Germany). The PGPR concentrations and W_1_-to-O volume ratios were screened in terms of emulsion appearance, micromorphology, and emulsion droplet size for the subsequent preparation of W_1_/O/W_2_ emulsions.

#### 2.3.2. Preparation of W_1_/O/W_2_ Pickering Emulsions

The PPSPI–PEC complex dispersion (with a 0.02 g/mL protein content; pH 3.0) was used as the continuous phase (W_2_), and the above emulsion was used as the dispersed phase. The W_1_/O emulsion was added to the PPSPI–PEC complex dispersion, and then the mixture was homogenized at 6000 rpm for 5 min. The mixture was then passed through a NS1001 L2K high-pressure homogenizer (GEA Niro Soavi S.p.A, Parma, Italy) at 5 MPa to obtain the emulsion.

The (W_1_/O)-to-W_2_ volume ratios were 2:8, 3:7, 4:6, 5:5, and 6:4 (*v*/*v*). The other operations were performed as above, and examinations were conducted to determine the optimum (W_1_/O)-to-W_2_ volume ratio.

#### 2.3.3. Observation of the Emulsions’ Microscopic Morphologies

The W_1_/O emulsions were examined using a BX53 light microscope with a 40× objective lens (Olympus China Co., Ltd., Tokyo, Japan), while the W_1_/O/W_2_ emulsions were examined using a fluorescence microscope with a 40× objective lens (Invitrogen EVOS FL Auto 2, Thermo Fisher Scientific, Carlsbad, CA, USA). Pipette 3 μL of emulsion on a slide, cover with a coverslip to make it spread evenly, and then observe it under the microscope.

#### 2.3.4. Droplet Size of W_1_/O Emulsions and W_1_/O/W_2_ Pickering Emulsions

For the W_1_/O emulsions, the droplet size of at least 40 emulsion droplets was determined using Image J 1.52v software, and the average droplet size was determined [45,46].

For the W_1_/O/W_2_ Pickering emulsions, the droplet size distribution and volume average droplet size were calculated using a Mastersizer 3000 Laser Diffraction Particle Size Analyzer (Malvern Instruments Ltd., Malvern, UK), with deionized water as the liquid medium.

#### 2.3.5. Determination of Encapsulation Efficiency (EE)

EE was measured based on the methodology reported by Lin et al. [38], with appropriate modifications. Place 1 mL of fresh emulsion, or emulsion that has been stored for a certain period of time, into a centrifuge tube, then add 2 mL of deionized water. Disperse the emulsion by shaking it well to ensure better dispersion of W_2_ in water. Centrifuge at 4000 r/min for 10 min. Then, carefully recover the water phase from the lower supernatant using a syringe and filter it through a 0.45 μm polyethersulfone (PES) filter. The concentration of anthocyanins in the lower supernatant was detected using the pH differential method [47]. Samples were diluted in aqueous pH 1.0 (0.025 M) and pH 4.5 (0.4 M) buffers, and the absorbance was measured at 520 nm and 700 nm. The anthocyanin content (C) was calculated and expressed as cyanidin-3-O-glucoside (C3G) equivalent according to the following equation:

(1) $$C\;(\mathrm{mg}/\mathrm{mL})=\frac{A\times Mw\times DF}{\varepsilon \times L}$$

A = (*A*_520_ – *A*_700_)*_pH_*_1.0_ – (*A*_520_ – *A*_700_)*_pH_*_4.5_ (2)

where *A*_520_ and *A*_700_ are absorbance at 520 and 700 nm, respectively; Mw is the molecular weight of C3G (449.2 g/mol); DF is the dilution factor; ε is the extinction coefficient (26,900 mol/L·cm^−1^); and L is the path length (1 cm).

*EE*(%) = (*AT* − *AI*)/*AT* × 100 (3)

where *AT* is the total quantity of anthocyanins added to the W_1_ phase, and *AI* is the initial amount of unencapsulated anthocyanins in the W_2_ phase after the preparation of the double emulsion.

At predetermined time points, *EE* values were determined using the procedure described above.

#### 2.3.6. Scanning Electron Microscopy (SEM)

According to Li et al. [48], the W_1_/O/W_2_ emulsion was lyophilized after storage at 4 °C for 24 h. The lyophilized samples were uniformly fixed on conductive adhesive tape and observed using an SU8010 scanning electron microscope (Hitachi, Tokyo, Japan). The images were captured using an accelerating voltage of 5 kV.

#### 2.3.7. Transmission Electron Microscopy (TEM)

The dispersion of the W_1_/O/W_2_ Pickering emulsion was blended in an equal volume of phosphotungstate (0.02 g/mL). The sample was photographed using an HT7820 transmission electron microscope (Hitachi, Tokyo, Japan).

### 2.4. Environmental Stability

#### 2.4.1. Thermal Stability

To explore whether the emulsion can maintain stability under variable temperature conditions, after referring to Zhang et al.’s research [49], the prepared W_1_/O/W_2_ Pickering emulsions were placed in a water bath at temperatures of 30 °C, 40 °C, 50 °C, 60 °C, 70 °C, and 80 °C for 30 min and then immediately cooled to room temperature. In this way, the thermal stability of the emulsions was evaluated.

#### 2.4.2. Effect of Ionic Strength on the Stability of the Emulsions

In order to investigate whether the emulsions can withstand varying ionic strengths, the concentrations of NaCl in the prepared W_1_/O/W_2_ Pickering emulsions were adjusted to 0, 50, 100, 200, and 500 mmol/L, with reference to the research by Qian et al. [50], and the emulsions were noted as 0, 50, 100, 200, and 500 mM to assess the effect of ionic strength on the stability of the emulsions.

#### 2.4.3. Freeze–Thaw Stability

Considering the findings of prior studies [51] and with the aim of investigating the emulsion’s ability to withstand multiple freeze–thaw cycles, the number of freeze–thaw cycles was established at 3. Newly formulated emulsions were subjected to one, two, and three freeze–thaw cycles, each consisting of 24 h at −30 °C followed by 2 h of thawing in a 20 °C water bath.

The effects of the above treatment conditions on the emulsions were evaluated in terms of the macroscopic behavior of the emulsions, emulsion droplet size, microscopic morphology, and anthocyanin retention. The antioxidant capacity of the anthocyanins in the emulsions after heat treatment was evaluated.

#### 2.4.4. Observation of Emulsions’ Microscopic Morphologies

The procedure carried out at this stage was the same as that reported in Section 2.3.3.

#### 2.4.5. Emulsion Droplet Size

The procedure carried out at this stage was the same as that reported in Section 2.3.4.

#### 2.4.6. Anthocyanin Retention

Refer to the method proposed by Tian et al. [52], with some modifications. A total of 1 mL of emulsion was added to 4 mL of anhydrous ethanol, vortexed, and sonicated for 10 min at 200 W. The supernatant was collected following centrifugation at 4000 r/min for 10 min. After filtration through a 0.45 μm filter membrane, the anthocyanin content was determined using the pH-differential method.

#### 2.4.7. Antioxidant Properties

##### DPPH

Make reference to the methodologies proposed by Zheng et al. [53] and Kucuk et al. [54], with appropriate modifications. A total of 0.1 mL of the sample extract solution was mixed with 1.9 mL of DPPH ethanol solution at a concentration of 0.1 mmol/L, and the volume was brought up to 4 mL with anhydrous ethanol. The mixture was then incubated for 30 min at room temperature, protected from light, and the absorbance value was measured at 517 nm. The absorbance, A_1_, was determined at 517 nm using the sample extract solution, whereas A_0_ was obtained under the same conditions using an equal volume of anhydrous ethanol as a blank. The following formula was used to compute the DPPH radical scavenging rate:

DPPH free radical scavenging rate (%) = (*A*_0_ – *A*_1_)/*A*_0_ × 100 (4)

##### ABTS

Utilizing the approach of Li et al. [55] as a basis, mix 7 mmol/L ABTS solution and 2.45 mmol/L potassium persulfate solution in equal volumes and incubate the mixture for 16 h to obtain the ABTS reserve solution. The ABTS reserve solution was diluted with ethanol to achieve an absorbance value of 0.700 ± 0.020 at 734 nm, thereby obtaining the ABTS working solution. Afterward, 0.1 mL of the test solution was blended with 3.9 mL of the ABTS working solution, and the mixture was incubated for 6 min at 734 nm to measure the absorbance value. The following formula was used to determine the ABTS free radical scavenging rate:

ABTS free radical scavenging rate (%) = (*A*_0_ – *A*_1_)/*A*_0_ × 100 (5)

where *A*_0_ is the absorbance value after incubation without the sample, and *A*_1_ is the absorbance value after incubation with the sample.

### 2.5. Statistical Analysis

All experiments were repeated at least 3 times. The results shown are averages ± standard deviations. One-way ANOVA (Duncan’s test (*p* < 0.05)) was used for significant difference analysis using SPSS 20.0 software.

## 3. Results and Discussion

### 3.1. Determination of W_1_-to-O Volume Ratio

**Figure 2:**
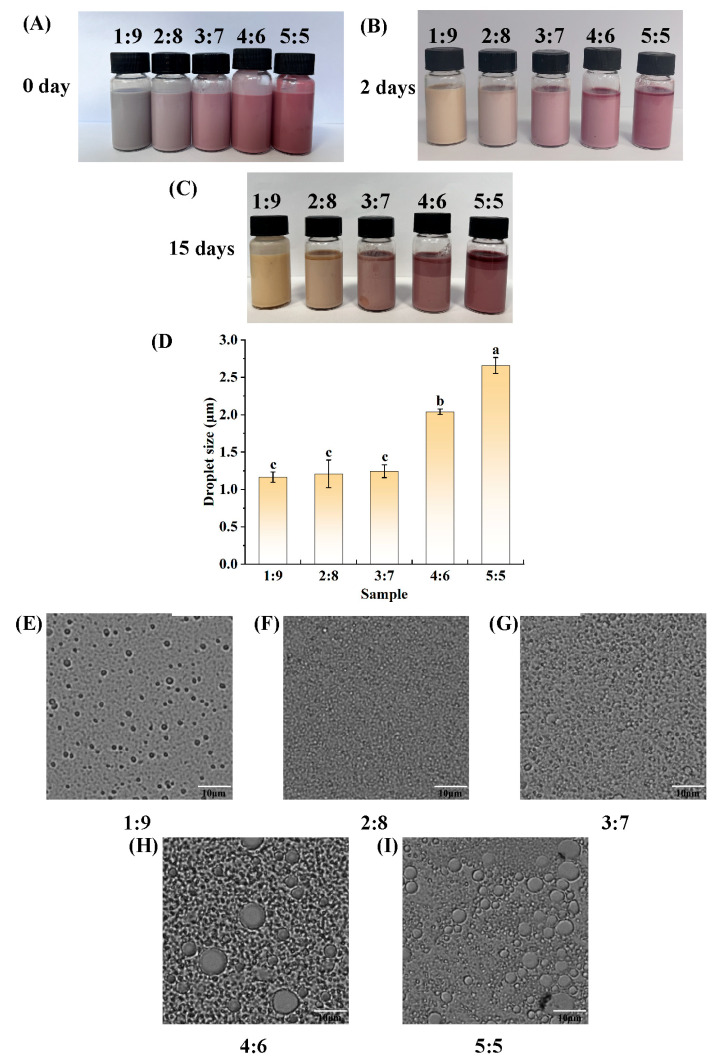
Visual appearance (**A**–**C**), average droplet size (**D**), and light microscope images (**E**–**I**) of W_1_/O emulsions at different volume ratios. Bars represent averages ± standard deviations. Different lowercase letters (a, b, c) indicate statistically significant differences between groups (one-way ANOVA followed by Duncan’s test (*p* < 0.05)). Groups labeled with the same letter do not differ significantly.

When the concentration of PGPR added was 5% of the oil phase volume, the W_1_-to-O volume ratios were set to 1:9, 2:8, 3:7, 4:6, and 5:5 for the production of a W_1_/O emulsion. The appearance of the prepared W_1_/O emulsion before and after 15 days of storage at room temperature (not protected from light) is shown in Figure 2A–C. The fresh W_1_/O emulsions loaded with anthocyanins were uniform in appearance, and due to the various W_1_-to-O volume ratios, their color varied slightly, gradually changing from grey to light pink and then to dark pink. After 2 days of storage at room temperature, all the emulsions were still homogeneous in appearance, but the colors had clearly changed, showing a certain degree of discoloration, probably due to the destruction of the anthocyanins. Fifteen days later, the emulsions with ratios of 4:6 and 5:5 showed obvious layering, indicating that the W_1_/O emulsions with W_1_-to-O volume ratios of 4:6 and 5:5 were unable to maintain relatively uniform and stabilized systems for a long period under room temperature conditions in a lipophilic emulsifier, PGPR, at 5% of the oil phase by volume. Moreover, Figure 2E–I and a comparison of the average droplet sizes of the emulsions could also reflect the differences among the W_1_/O emulsions prepared with different W_1_-to-O volume ratios. The microscopic morphologies of the W_1_/O emulsions are presented in Figure 2E–I. Each sample had almost spherical water droplets scattered throughout the oil phase, but quite a few droplets with larger droplet sizes could clearly be seen in the W_1_/O emulsions with W_1_-to-O volume ratios of 4:6 and 5:5. As shown in Figure 2D, the droplet size of the W_1_/O emulsion with a W_1_-to-O volume ratio of 5:5 was the largest, on average, amounting to 2.66 μm. The droplet size of the W_1_/O emulsion with a W_1_-to-O volume ratio of 4:6 was the second largest, amounting to 2.04 μm. The droplet sizes of the W_1_/O emulsions with W_1_-to-O volume ratios of 1:9, 2:8, and 3:7 did not differ much from each other, with sizes of 1.16, 1.21, and 1.24 μm, respectively. When the W_1_-to-O volume ratio was 3:7, the emulsion could encapsulate relatively more anthocyanins and was stable, and no delamination occurred after 15 days of storage. Accordingly, a W_1_-to-O volume ratio of 3:7 was used in the subsequent experiments. In the experiment by Koirala et al. [4], they also prepared a W_1_/O emulsion encapsulating anthocyanin-rich butterfly pea petal extract with a W_1_-to-O volume ratio (3:7). The W_1_/O/W_2_ emulsion they subsequently prepared exhibited better stability and could remain stable for 7 days. Rabelo et al. [56] also prepared stable nano W/O emulsions encapsulating açaí berry anthocyanins using tetraglycerin monolaurate condensed ricinoleic acid esters when the dispersed phase weight fraction was 30 wt%.

### 3.2. Determination of the Amount of PGPR

**Figure 3:**
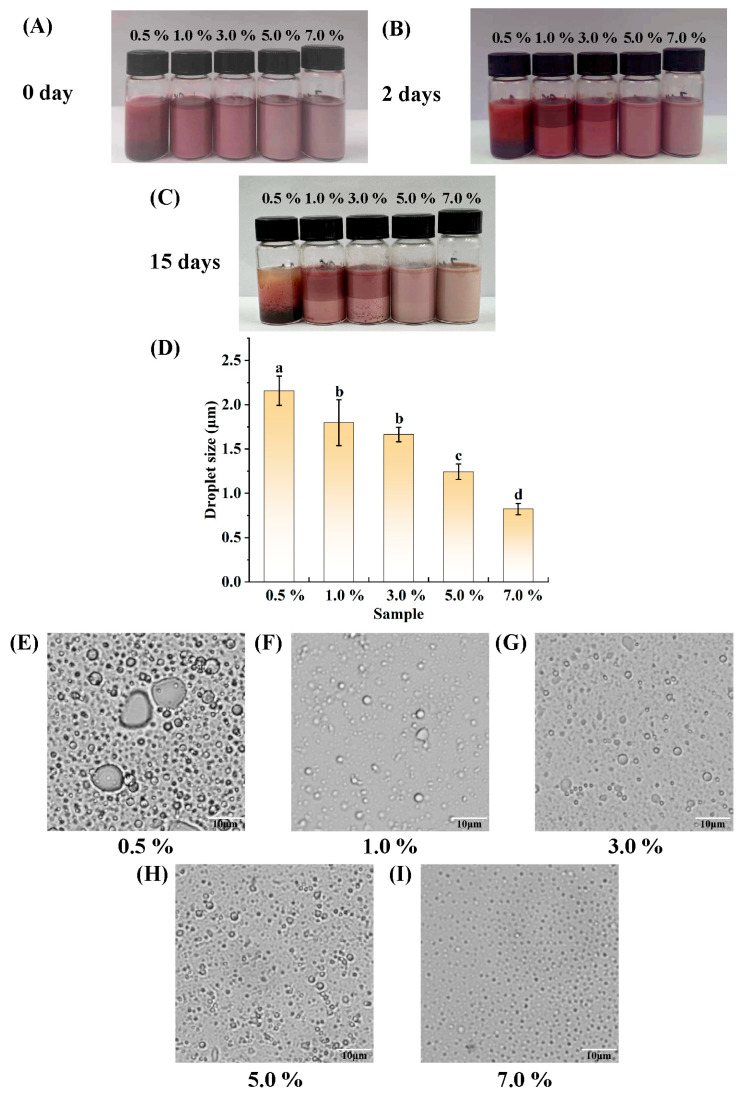
Visual appearance (**A**–**C**), average droplet size (**D**), and light microscope images (**E**–**I**) of W_1_/O emulsions with different PGPR additions. Bars represent averages ± standard deviations. Different lowercase letters (a, b, c, d) indicate statistically significant differences between groups (one-way ANOVA followed by Duncan’s test (*p* < 0.05)). Groups labeled with the same letter do not differ significantly.

The W_1_/O emulsions were produced by setting the concentrations of the lipophilic emulsifier, PGPR, to 0.5%, 1.0%, 3.0%, 5.0%, and 7.0% of the volume of the oil phase when the W_1_-to-O volume ratio of the emulsion was 3:7. The appearance of the prepared W_1_/O emulsion before and after storage for 15 days at room temperature (not protected from light) is shown in Figure 3A–C. Evidently, the freshly prepared W_1_/O emulsions with PGPR concentrations of 1.0%, 3.0%, 5.0%, and 7.0% have a uniform appearance, but the W_1_/O emulsion at 0.5% PGPR started to stratify, and the anthocyanin solution deposited in the lower layer can be seen. At the same time, the color of each treatment group is inconsistent, ranging from red to pink to light pink, which could be due to the different amounts of PGPR added and the different encapsulation efficiencies of the emulsions for the anthocyanins, with the content of unencapsulated anthocyanin solutions decreasing gradually, resulting in the colors changing from darker to lighter. After storage at room temperature for 2 days, the W_1_/O emulsions with PGPR concentrations of 0.5%, 1.0%, and 3.0% all showed some degree of stratification. After 15 days of storage at room temperature, the W_1_/O emulsions at 0.5%, 1.0%, and 3.0% PGPR showed some degree of layering, suggesting that the W_1_/O emulsions prepared under these conditions were unsuitable for long-term storage, whereas the W_1_/O emulsions with PGPR concentrations of 5.0% and 7.0% did not show significant delamination. After 15 days of storage at room temperature, all the samples showed some degree of discoloration. The microscopic morphologies (Figure 3E–I) of the emulsions from all the treatment groups revealed nearly spherical droplets inside. Droplet sizes decreased roughly as the concentration of PGPR increased. The mean droplet size of the emulsions (Figure 3D) and the microscopic results (Figure 3E–I) are consistent, showing that the droplet size becomes smaller upon increasing PGPR concentration. In the W_1/_O emulsions stabilized by PGPR prepared by Wu et al. [57], as the concentration of PGPR varied from 1%, 3%, 5% to 7%, the droplet size of the emulsions decreased accordingly, and only the emulsion with 7% PGPR remained stable during 14 days of storage. In the study conducted by Lin et al. [38], the encapsulation efficiency of anthocyanins in the W_1_/O/W_2_ emulsions they prepared increased as the PGPR concentration rose, and there was little difference in encapsulation efficiency at PGPR concentrations of 2%, 4%, and 6%. Considering both the amount of PGPR used and the aforementioned experimental results, it was determined that the amount of PGPR added should be 5% of the oil phase by volume.

### 3.3. Determination of the (W_1_/O)-to-W_2_ Volume Ratio

**Figure 4:**
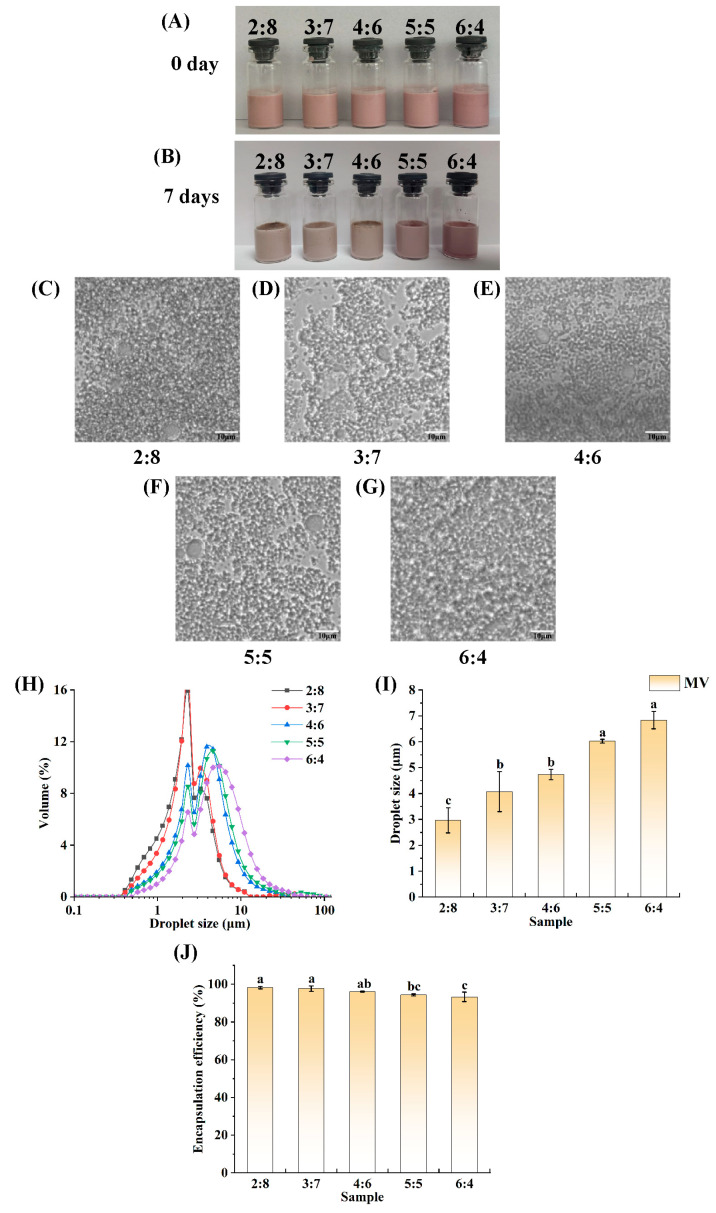
Visual appearance (**A**,**B**), fluorescence microscopic images (**C**–**G**), droplet size distributions (**H**), average droplet size (**I**), and encapsulation efficiency (**J**) of W_1_/O/W_2_ emulsions with different W_1_/O-to-W_2_ volume ratios. Bars represent averages ± standard deviations. Different lowercase letters (a, b, c) indicate statistically significant differences between groups (one-way ANOVA followed by Duncan’s test (*p* < 0.05)). Groups labeled with the same letter do not differ significantly (e.g., a and ab share the letter “a”, so they are not significantly different).

To prepare W_1_/O/W_2_ emulsions, it is first necessary to prepare stable W_1_/O emulsions. Since the presence of preservatives may affect the interfacial properties of emulsions [58], a zero-addition protocol was chosen to keep the variables under control in order to completely eliminate the interference of preservatives. Notably, for some ready-to-eat foods, a storage period of 7 days is sufficient. In this experiment, the W_1_/O/W_2_ emulsions were stored at room temperature for 7 days. The W_1_/O emulsions had to be stored for 7 days or longer, so a storage period of 15 days was set for the W_1_/O emulsions. Figure 4A and B show the macromorphologies of the emulsions with different (W_1_/O)-to-W_2_ volume ratios. The emulsions were stored at room temperature for 7 days. The freshly prepared emulsions were in a homogeneous state without delamination. The color of the emulsions changed from light pink to pink depending on the (W_1_/O)-to-W_2_ volume ratios. After 7 days of storage at room temperature, the states of the emulsions were stable, with no macroscopic destabilization. Overall, the paleness of the emulsions was attributed to the decomposition of anthocyanins during storage.

Figure 4C–G show the micromorphologies of the emulsions with different (W_1_/O)-to-W_2_ volume ratios. As shown in all the Figures, the emulsion droplets exhibited an intensified flocculation phenomenon as the proportion of W_1_/O in the double emulsion increased. Although the emulsions exhibited flocculation, they still tended to be stable overall.

Figure 4H shows the droplet sizes of the W_1_/O/W_2_ emulsions with different (W_1_/O)-to-W_2_ ratios. The droplet size of each sample roughly shows a single-peaked distribution. The droplet size of the emulsions shifted toward larger sizes with increasing (W_1_/O)-to-W_2_ volume ratio. Notably, in Figure 4I, the average droplet sizes of the different emulsions gradually increased with an increase in the (W_1_/O)-to-W_2_ volume ratio. The smallest droplet, 2.97 μm, was found at a ratio of 2:8, and the largest droplet, 6.83 μm, was found at 6:4. Contrary to the aforementioned conclusion, Huang et al. [37] observed that the droplet size of the double emulsion they prepared decreased with an increase in the volume fraction of the W_1_/O emulsion. The primary reason for this discrepancy is that gelatin was not added to the W_1_ phase in the our study, which allowed the droplet movement to be unrestricted by a gel-like network structure. Compared to the double emulsion droplet size (greater than 10 μm) in the study conducted by Wu et al. [57], the droplet size of the emulsion in this research is relatively smaller, generally less than 10 μm. The reason for this result may be that the emulsion was subjected to treatment by a high-pressure homogenizer.

Figure 4J shows the EE of the different freshly made samples. The EE was calculated for emulsions with different (W_1_/O)-to-W_2_ volume ratios. The results show that the PPSPI–PEC complexes at all ratios created enough of a barrier at the O–W_2_ phase interface to lock most of the anthocyanins in the double emulsion. As shown in Figure 4J, the greatest emulsion EE, 98.22%, was observed when the (W_1_/O)-to-W_2_ volume ratio was 2:8. The lowest emulsion EE, 93.29%, was observed when the (W_1_/O)-to-W_2_ volume ratio was 6:4. The EE of the remaining samples ranged from 94.39% to 97.71%. At a ratio of 3:7, the EE was at the same level of significance as that at a ratio of 2:8. Compared with the research results presented in this paper, the EE (encapsulation efficiency) of the double emulsions prepared by Jiang et al. [59] was less than 80%. The double emulsions prepared by Lin et al. [38] achieved the maximum EE at a (W_1_/O)-to-W_2_ volume ratio of 6:4 and 5:5, with an approximate value of 97%.

When the (W_1_/O)-to-W_2_ volume ratio was 3:7, the emulsion droplets were smaller in size and exhibited a higher EE. Compared with the emulsion that had a (W_1_/O)-to-W_2_ volume ratio of 2:8, the emulsion with a (W_1_/O)-to-W_2_ volume ratio of 3:7 could encapsulate more of the anthocyanin solution. In summary, the (W_1_/O)-to-W_2_ volume ratio was determined to be 3:7.

### 3.4. SEM and TEM

**Figure 5:**
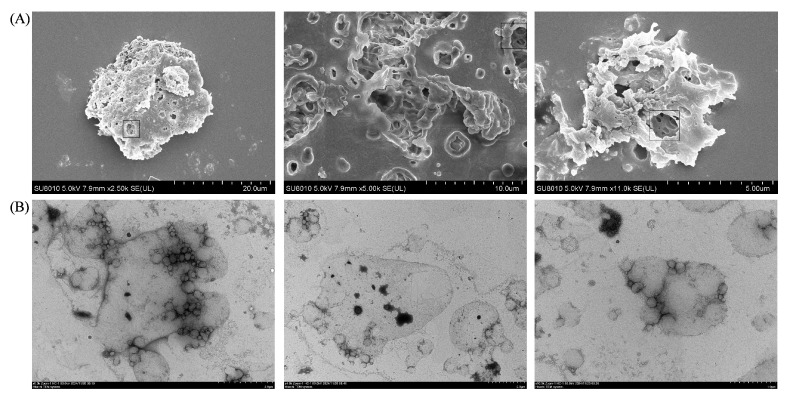
Scanning electron microscopy images (**A**) and transmission electron microscopy images (**B**) of anthocyanin-loaded double Pickering emulsions stabilized by PPSPI–PEC complexes with 5% (*v*/*v* of oil) PGPR, a 2.0 wt% PPSPI concentration, and (W_1_/O = 3:7):W_2_ = 3:7.

SEM and TEM were used to observe the morphologies of the samples [60,61,62]. The SEM results show the presence of internal voids in the freeze-dried W_1_/O/W_2_ emulsion due to inner water sublimation (Figure 5A). This phenomenon is similar to what was observed in the studies by Li et al. [48] and Liang et al. [63]. The presence of small droplets in large droplets, as shown in Figure 5B, proved the existence of a double-emulsion structure in the emulsion, and this microstructure was similar to what was observed in the studies by Choi et al. [64] and Silva et al. [65]. The W_1_ droplets within a freshly made W_1_/O/W_2_ double emulsion should be evenly distributed in the oil phase, with similar morphologies and sizes. However, a larger W_1_ droplet appeared in a TEM image presented by Silva et al. This droplet may have formed because of the flocculation of the inner water phase (W_1_) droplet over time and because the emulsion itself is a thermodynamically unstable system.

### 3.5. Thermal Stability

**Figure 6:**
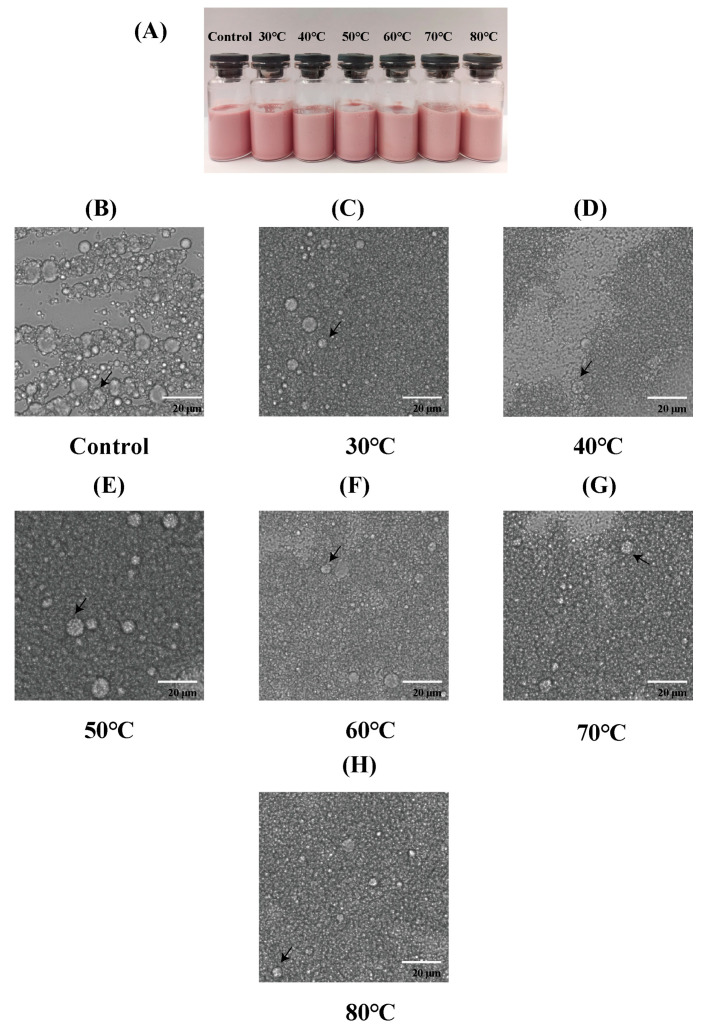
Visual appearance (**A**) and fluorescence microscope images (**B**–**H**) of W_1_/O/W_2_ emulsions at different treatment temperatures. The arrows denote double emulsion droplets.

Heat treatment is widely recognized as a key approach for enhancing or developing the flavor of food matrices that are rich in proteins and lipids. Consequently, various cooking techniques, such as baking, steaming, and microwaving, have been employed to optimize the physical, textural, quality, and flavor attributes of foods [66,67]. Meanwhile, heat treatment is also one of the most common methods for sterilization [68,69]. Therefore, it is necessary to determine the heat treatment properties of emulsions for their further utilization in food. As shown in Figure 6A, the morphologies of the emulsions changed after heat treatment at different temperatures. Compared with the control group (which was not treated), the color of the emulsion became lighter. Basically, the higher the temperature, the lighter the color of the emulsion. This is likely the result of the destruction of anthocyanins in the emulsions. In addition, after heat treatment, the emulsions did not undergo delamination at each treatment temperature, indicating that the emulsions exhibited thermal stability to some extent. Previous studies have indicated that anthocyanin-loaded double Pickering emulsions stabilized with β-cyclodextrin exhibit different states after 30 min of water bath treatment at 50 °C, 70 °C, and 95 °C. The emulsions at 50 °C and 70 °C showed different degrees of decoloration, and significant delamination occurred in the emulsions treated at 95 °C [11].

Figure 6B–H show the microscopic morphologies of the emulsions at different heat treatment temperatures. All figures show that the emulsions had a dual structure, with the droplets remaining inside the oil droplets. The microstructures of the emulsions remained unchanged at all heat treatment temperatures, further proving the superior thermal stability of these emulsions.

**Figure 7:**
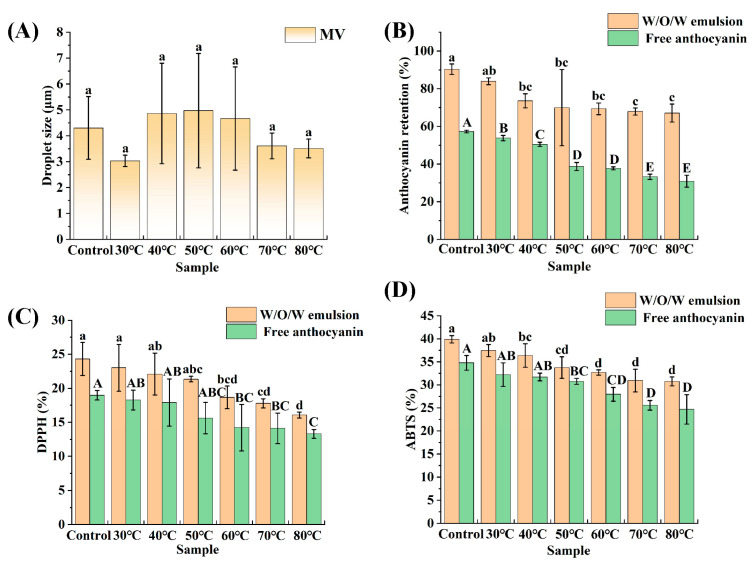
Average droplet size (**A**), anthocyanin retention (**B**), DPPH radical scavenging activity (**C**), and ABTS radical scavenging activity (**D**) of W_1_/O/W_2_ emulsions at different treatment temperatures. The green bars represent the blank control group in the experiment, which is the anthocyanin solution without encapsulation. Bars represent averages ± standard deviations. Different letters (a, b, c, d; A, B, C, D, E) indicate statistically significant differences between groups (one-way ANOVA followed by Duncan’s test (*p* < 0.05)). Groups labeled with the same letter do not differ significantly.

In addition, the average droplet sizes of the emulsions at different heat treatment temperatures were recorded (Figure 7A). According to a comparative analysis, the average droplet sizes among the samples did not exhibit significant variability, with sizes ranging from approximately 3.03 to 4.97 μm. It can be observed from the figure that the standard deviation of the average droplet size of the emulsion initially increases and then decreases, with relatively large standard deviations at 40 °C, 50 °C, and 60 °C. This result may be attributed to a combination of multiple factors. The impact of heat treatment alone on the emulsifying capacity of proteins can yield two opposing outcomes: it may expose hydrophobic groups of proteins, leading to protein unfolding and a reduction in interfacial tension, which enhances the emulsifying capacity; alternatively, it may induce unfavorable conformational changes in proteins, such as aggregation and masking of hydrophobic sites, thereby reducing the emulsifying capacity [70]. When analyzing solely the trend in the standard deviation of emulsion droplet size, it increased at first, and then it decreased with rising temperature in the experiment. This suggests that heat treatment at 40 °C, 50 °C, and 60 °C may reduce the emulsifying capacity of proteins. Meanwhile, given the relatively low heat treatment temperatures of 40 °C, 50 °C, and 60 °C, and considering that double emulsions represent a relatively complex system, proteins may experience varying degrees of heat treatment, leading to significant differences in their emulsifying capacities. Consequently, there are substantial variations in emulsion droplet sizes and larger standard deviations in the droplet size distribution. In contrast, at 70 °C and 80 °C, the emulsifying capacity of proteins may be enhanced, and coupled with relatively higher heat treatment temperatures, proteins are subjected to more uniform heat treatment conditions, resulting in smaller standard deviations in emulsion droplet size at these temperatures.

Figure 7B demonstrates the anthocyanin retention of the emulsions at different heat treatment temperatures. Overall, the anthocyanin retention of the emulsions shows a trend of decreasing as the heat treatment temperature increases. The anthocyanin retention of the newly prepared emulsion was 90.37%, followed by 83.95%, 73.57%, 69.96%, 69.32%, 67.91%, and 67.09%, respectively. The above phenomenon can be attributed to the poor thermal stability of anthocyanins. The higher the heat treatment temperature, the greater the loss of anthocyanin and the lower the amount retained. During heat treatment at 30–40 °C, the anthocyanin retention in all groups was always higher than 70%, indicating that the double emulsion had a strong protective effect on the thermal degradation of anthocyanins at low temperatures. The anthocyanin retention of the double emulsions was higher than 65% after heat treatment at 50–80 °C, indicating that the double-emulsion structure could provide effective protection for anthocyanins. The thermal stability of zein–anthocyanin nanoparticles was investigated in the study by Li et al. [71]. Under heat treatment conditions at temperatures of 60, 70, 80, and 90 °C, the residual rates of anthocyanins also decreased roughly in line with the increase in treatment temperature.

Anthocyanins are water-soluble pigments widely available in nature [72]. These compounds are powerful antioxidants and possess a range of biological activities, such as antioxidant, anti-inflammatory, immune-regulating, and growth-promoting activities in animals. Their antioxidant capabilities primarily involve the scavenging of reactive oxygen species, the neutralization of free radicals, and the activation of antioxidant enzyme pathways [73]. It is essential to determine the antioxidant properties of anthocyanins. In this study, the protective effect of W_1_/O/W_2_ emulsions on the antioxidant properties of anthocyanins was evaluated by determining DPPH and ABTS free radical scavenging rates. The antioxidant properties of anthocyanins in emulsions after subjection to heat treatment at different temperatures for 30 min are shown in Figure 7C,D. The initial DPPH radical scavenging activity of anthocyanins in the emulsion was 24.30%, and the initial ABTS radical scavenging activity was 39.90%. Evidently, the scavenging activity of ABTS radicals exhibited by anthocyanins within the emulsion is markedly superior. As the heat treatment temperature increased, both the DPPH and ABTS radical scavenging activities of the emulsion anthocyanins declined, with similar degrees of reduction. The decreases in DPPH and ABTS radical scavenging activity were approximately 8% and 9%, respectively. The results indicate that the W_1_/O/W_2_ emulsions stabilized by the PPSPI–PEC complexes significantly slowed the decrease in the antioxidant properties of the anthocyanins after being subjected to heat treatments at different temperatures, an outcome related to the fact that a relatively high anthocyanin content was retained in the emulsions after being subjected to heat treatments. The slower decrease in the antioxidant properties of anthocyanins in the W_1_/O/W_2_ emulsions may be attributed to the fact that the double emulsions have two oil–water interfacial membranes, which provide a good barrier against oxygen [74].

### 3.6. Effect of Ionic Strength on the Stability of the Emulsions

**Figure 8:**
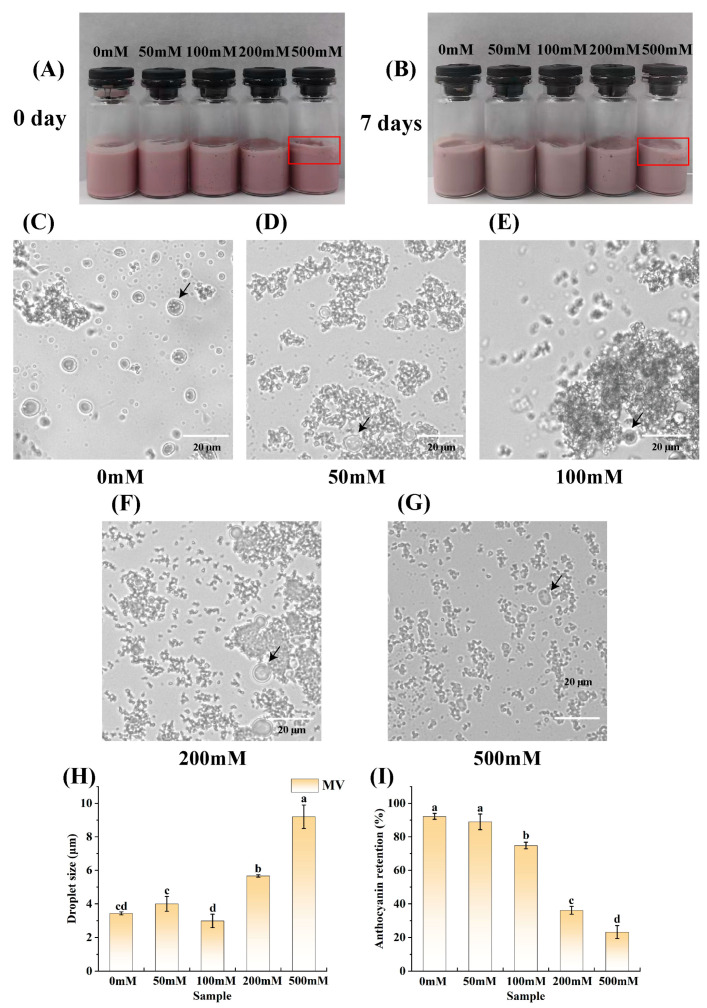
Visual appearance (**A**,**B**), fluorescence microscopic images (**C**–**G**), average droplet size (**H**), and anthocyanin retention (**I**) of W_1_/O/W_2_ emulsions at different ionic strengths. The arrows denote double emulsion droplets, while the area within the solid red square is the flocculated region of the emulsion. Bars represent averages ± standard deviations. Different lowercase letters (a, b, c, d) indicate statistically significant differences between groups (one-way ANOVA followed by Duncan’s test (*p* < 0.05)). Groups labeled with the same letter do not differ significantly.

The ionic strength of emulsions is important for their stability [75]. Within a specific range of ionic strength, increasing ionic strength can modify the charge distribution on the surfaces of protein molecules. This change subsequently affects the electrostatic interactions between protein molecules, promoting protein unfolding and gel formation [76]. However, when the ionic strength becomes excessively high, the diminished repulsive forces between proteins cannot counteract the attractive forces, resulting in the aggregation of oil droplets. Additionally, ions can either enhance or inhibit the stabilization of emulsions by influencing the aggregation and adsorption behaviors of plant proteins, such as soy and corn proteins [77]. The surface charges of solid particles, as well as ion–dipole and ion–ion interactions in solution, are altered by pH and ionic strength, which, in turn, affect the interfacial properties and emulsion stability of Pickering particles [78]. Therefore, in this study, the effect of ionic strength on the stability of emulsions was investigated. As shown in Figure 8A,B, which present macroscopic physical images of the emulsions at different ionic strengths, the area marked by the red box in Figure 8A,B reveals that the sample with an ionic strength of 500 mM showed flocculation. Compared to the freshly prepared emulsion samples, the samples stored at room temperature for 7 days were slightly lighter in color due to the degradation of anthocyanins within the emulsion.

Figure 8C–G show the microscopic morphologies of the emulsions at different ionic strengths. As shown in Figure 8C–G, the emulsions exhibited a dual structure, with water droplets encapsulated within oil droplets.

Figure 8H shows the average droplet sizes of the emulsions at different ionic strengths. Evidently, the average droplet sizes of the samples with ionic strengths of 50 mM and 100 mM did not differ significantly from the average droplet size of the untreated control (3.43 μm), whose droplets were determined to be 4.00 μm and 2.99 μm, respectively. The droplets of the emulsion with an ionic strength of 100 mM were smaller than those of the emulsion with an ionic strength of 50 mM. The reason for this phenomenon might be that the addition of a certain concentration of NaCl to the outer aqueous phase (W_2_) facilitated the balancing of the osmotic pressure between the inner aqueous phase (W_1_) and the outer aqueous phase and prevented the migration of water [79,80]. When the ionic strength reached 200 mM, the emulsion droplet size significantly deviated from that of the control, being notably larger, at 5.66 μm. Furthermore, when the ionic strength of the emulsion was increased to 500 mM, the emulsion droplet size further increased to 9.2 μm. The substantial increase in emulsion droplet sizes observed at ionic strengths of 200 mM and 500 mM could be explained by the accumulation of charged ions around the PPSPI–PEC complex. These ions neutralized the surface charge of the complex, thereby reducing the electrostatic repulsion between droplets. Consequently, the droplets were more likely to coalesce, making them larger [81,82].

Figure 8I shows the anthocyanin retention of the emulsions at different ionic strengths. Evidently, the anthocyanin retention in the emulsions generally exhibited a trend of decreasing with an increase in ionic strength. The newly prepared emulsion had an anthocyanin retention of 92.21%. Then, the anthocyanin retention for each subsequent group decreased sequentially, being 88.98%, 74.80%, 36.16%, and 23.22%. Under ionic strengths ranging from 50 to 100 mM, the anthocyanin retention consistently remained above 70%, indicating that the double-emulsion structure offered a certain degree of protection to the anthocyanins. In the study conducted by Feng et al. [83], the utilization of carboxymethyl cellulose (CMC) in conjunction with soybean protein isolate (SPI) was explored for stabilizing emulsions intended for β-carotene transportation. The β-carotene retention rate was observed to decrease approximately with an increase in ionic strength when tested at concentrations of 0, 50, 100, and 150 mM. It was postulated that, at ionic strengths of up to 200 mM, salt ions might exert a competitive influence on the electrostatic interactions in the SPI–CMC system, which acts as the emulsion stabilizer. Consequently, this led to the disruption of the interfacial properties in the SPI–CMC system, accompanied by a decline in their adsorption capacity at the interface of the emulsion droplets. Eventually, the disruption of the emulsion’s stability accelerated the degradation of β-carotene.

### 3.7. Freeze–Thaw Stability

**Figure 9:**
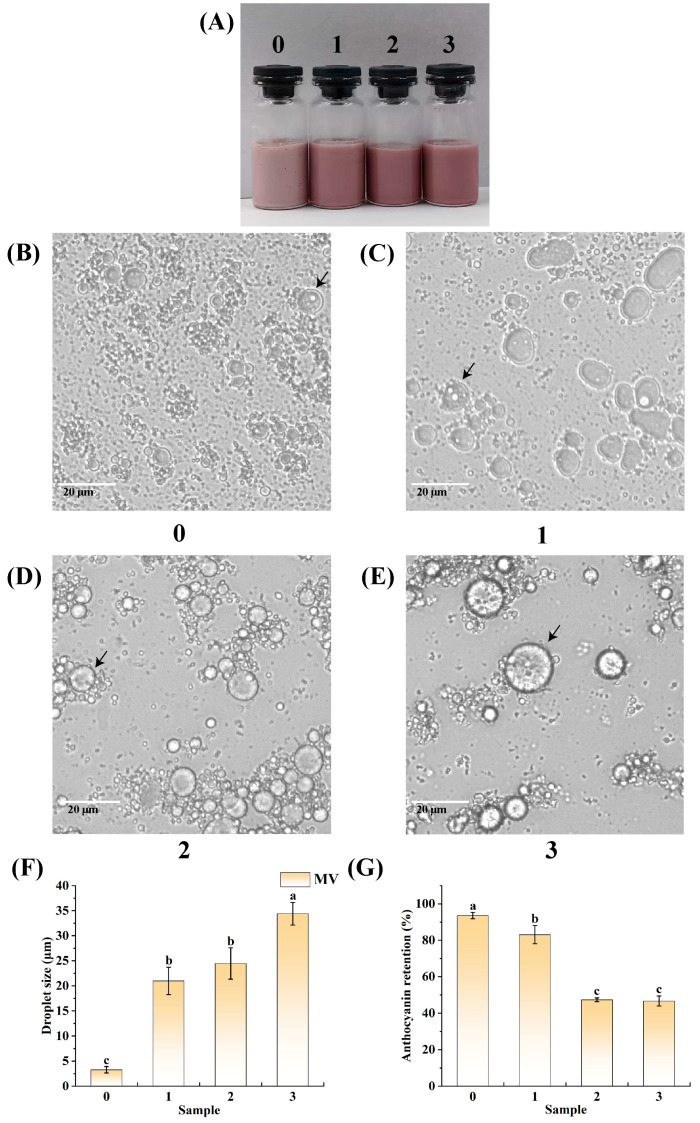
Visual appearance (**A**), fluorescence microscope images (**B**–**E**), average droplet size (**F**), and anthocyanin retention (**G**) of the W_1_/O/W_2_ emulsion under different freeze–thaw cycles. The arrows denote double emulsion droplets. Bars represent averages ± standard deviations. Different lowercase letters (a, b, c) indicate statistically significant differences between groups (one-way ANOVA followed by Duncan’s test (*p* < 0.05)). Groups labeled with the same letter do not differ significantly. The numbers 0, 1, 2, and 3 represent the number of freeze–thaw cycles the emulsion underwent.

Freezing is widely used as a food preservation method in ready-to-eat foods to extend shelf life by inhibiting microbial growth [84]. It usually requires thawing by conventional heating or microwaving before consumption. However, the freezing and thawing process usually leads to a reduction in the quality of the food [85]. Therefore, it is important for emulsions to have good freeze–thaw stability. Figure 9A shows a macroscopic physical picture of the emulsion after the freeze–thaw process. Evidently, compared with the untreated frozen and thawed samples, the color of the treated frozen and thawed samples was significantly deeper, and, essentially, the color of the samples became darker and darker as the samples underwent more freeze–thaw cycles, which may be attributed to the fact that, after the freeze–thaw cycles, the anthocyanins that were encapsulated in the emulsions leaked out, resulting in a deeper color of the emulsions. Figure 9A shows that the emulsion did not show obvious delamination after three freeze–thaw cycles.

Figure 9B–E show the microscopic morphologies of the emulsions subjected to different freeze–thaw cycles. As shown in Figure 9B–E, the emulsions had a dual structure, with the droplets remaining within the oil droplets. The emulsions still maintained the dual structure after different freeze–thaw cycle treatments. As shown in Figure 9B–E, the emulsion droplets became larger with the increase in the number of freeze–thaw cycles to which the sample was subjected. In the freeze–thaw experiments conducted by Zhang et al. [86], it was observed that the soy protein isolate–maltose-stabilized Pickering emulsions exhibited an escalating trend of droplet aggregation and flocculation as the number of freeze–thaw cycles increased. This may have been due to ice crystals causing damage to the protein membrane at the emulsified droplet interface. Consequently, the repeated cycles of freezing and thawing resulted in progressive flocculation of the droplets. When this flocculation reached a critical level, the soy protein isolate–maltose-stabilized Pickering emulsion underwent significant flocculation due to the tight aggregation or compression of the droplets.

Figure 9F shows the average droplet sizes of the emulsions under different freeze–thaw cycles. Evidently, the emulsion droplet sizes gradually increased with the increase in the number of freeze–thaw cycles experienced, in the order of 3.28 μm, 20.98 μm, 24.45 μm, and 34.40 μm. This result is consistent with the microscopic morphologies of the emulsions. In the study by Hong et al. [87], during freeze–thaw experiments that were conducted on high-internal-phase emulsions stabilized by trehalose and myofibrillar proteins, it was observed that the emulsion droplet size increased as the number of freeze–thaw cycles increased. They speculated that the ice crystals that had formed repeatedly punctured the boundary layer of the emulsion, forcing droplet flocculation and coalescence.

In Figure 9G, the anthocyanin retention of the emulsions after being subjected to different freeze–thaw cycles are documented in detail. With more freeze–thaw cycles, the anthocyanin retention of the emulsion decreased gradually, with values of 93.55%, 83.06%, 47.35%, and 46.70%. This may be due to the disruption of the emulsion’s stability during the freeze–thaw process. The experiments demonstrated that the emulsion possessed a certain degree of freeze–thaw stability. Notably, Lu et al. [88] used high-temperature glycation-modified egg white protein to stabilize high-internal-phase emulsions and found that the amount of encapsulated β-carotene in the emulsion decreased after undergoing three freeze–thaw cycles.

## 4. Conclusions

In this study, PPSPI–PEC complexes were prepared, and an investigation into the environmental stability of the double emulsions developed was conducted. The results revealed that the double emulsions were successfully formulated, and their stability was influenced by factors such as ambient temperature, ionic strength, and freeze–thaw treatment. The experimental data indicated that the emulsions exhibited greater stability when W_1_-to-O volume ratio and (W_1_/O)-to-W_2_ volume ratio were both 3:7. Following heat treatment at varying temperatures for 30 min, the emulsions’ microstructures remained relatively unchanged; however, anthocyanin retention decreased with an increase in treatment temperature, accompanied by alterations in the emulsions’ appearance. Ionic strength had an impact on the emulsions’ appearance, with the samples at an ionic strength of 500 mM exhibiting flocculation. Droplet sizes generally increased with a rising ionic strength, while anthocyanin retention decreased. Freeze–thaw treatment altered the state of the emulsions. The more freeze–thaw cycles the emulsions underwent, the more unstable they became, with larger droplet sizes and lower anthocyanin content. The findings demonstrate that the double emulsions prepared in this experiment exhibited some stability during environmental changes and storage, highlighting the significant potential of double emulsions stabilized by PSPI for the encapsulation of bioactives.

## Data Availability

The original contributions presented in the study are included in the article, further inquiries can be directed to the corresponding author.
